# Surgical Management of a Giant Adrenal Pseudocyst: A Case Report and Review of the Literature in the Last Decade

**DOI:** 10.1155/2018/8473231

**Published:** 2018-02-01

**Authors:** Daniel Paramythiotis, Petros Bangeas, Anestis Karakatsanis, Patroklos Goulas, Irini Nikolaou, Vasileios Rafailidis, Konstantinos Kouskouras, Vasileios Papadopoulos, Sofia Lypiridou, Georgia Karayannopoulou, Antonios Michalopoulos

**Affiliations:** ^1^1st Propedeutic Surgical Department, AHEPA University Hospital, Aristotle University of Thessaloniki, Thessaloniki, Greece; ^2^Department of Radiology, AHEPA University Hospital of Thessaloniki, Thessaloniki, Greece; ^3^Department of Pathology, AHEPA University Hospital of Thessaloniki, Thessaloniki, Greece

## Abstract

Adrenal pseudocysts are rare entities and occurred in the 5th and the 6th decades of life. They are discovered accidentally, while appearing with nonspecific clinical and imaging findings. We report a case of a 28-year-old woman presented in our Emergency Department complaining about upper abdomen pain. Computed tomography revealed a hypodense cystic lesion containing hyperdense material. The size of a mass was 11. 7 × 9.3 × 6.6 cm in diameter close to the pancreas, but the origin was from the left adrenal gland. The mass was excised with surgical laparotomy. Giant adrenal pseudocysts are rare entities. Final diagnosis usually confirmed with the pathology examination. Management of such adrenal lesions depends on the unique characteristics, the surgeon's experience, and local resources.

## 1. Introduction

The Viennese anatomist Greiselius was the first who described in 1670 a ruptured adrenal cyst, filled with 12 pounds of fluid and 2 pounds of clot, as the cause of death in a 45-year-old nobleman [1]. In general, cystic lesions of the adrenal gland are uncommon (0.064–0.18% in autopsy series) [2] and may demonstrate a variety of histological changes, from pseudocysts to malignant cystic neoplasms, representing just under 6% of all newly discovered incidentalomas [3]. Most adrenal cysts are small in size, and they may be bilateral in 10% of the cases. They are reported two to three times more frequently in women than in men, and they are usually diagnosed during the 5th and the 6th decades of life, without however an apparent etiology for the sex predilection [4].

According to the Levison classification [5], which originally classified the adrenal cysts into subtypes, adrenal cysts are distinguished into parasitic cysts, caused mostly by *Echinococcus* species; true glandular or retention cysts; lymphangiectatic cysts or cystic hemangiomas, due to ectasia of preexisting vessels; and finally, pseudocysts [5]. Nowadays, cystic lesions of the adrenal gland are divided into four groups: parasitic; epithelial (true cysts); endothelial (vascular cysts with an endothelial lining); and pseudocysts [6]. The pseudocysts are characterized by the absence of any endothelial or epithelial lining; they are enclosed by a fibrous wall and filled with fresh or altered blood [7]. Cystic lesions of adrenal glands are represented with nonspecific clinical and imaging findings, while diagnosis of the adrenal cyst is usually incidental [7, 8]. Imaging studies included abdominal computed tomography (CT), magnetic resonance imaging (MRI), and ultrasonography (US). The adrenal pseudocyst exhibits mixed characteristics on CT imaging, while on MRI images, it exhibits high density in T1 images and marked light up in T2 images. Diagnosis of adrenal pseudocysts is usually difficult and definitive on surgery. Herein, we describe a case of an adrenal pseudocyst in a young woman that was treated surgically in our department after investigation for upper abdominal pain. A review of the literature in the last decade considering this rare entity is also performed.

## 2. Case Report

A 28-year-old woman presented in the Emergency Department complaining about upper abdominal pain during the previous three days, without fever or anorexia, not related to food intake, and without bowel function abnormalities. Her surgical and clinical history was clear, and she had not noticed occurrence of the same symptoms before. During clinical examination, a palpable mass in the lower epigastric area was found, with focal tenderness.

**Figure 1:**
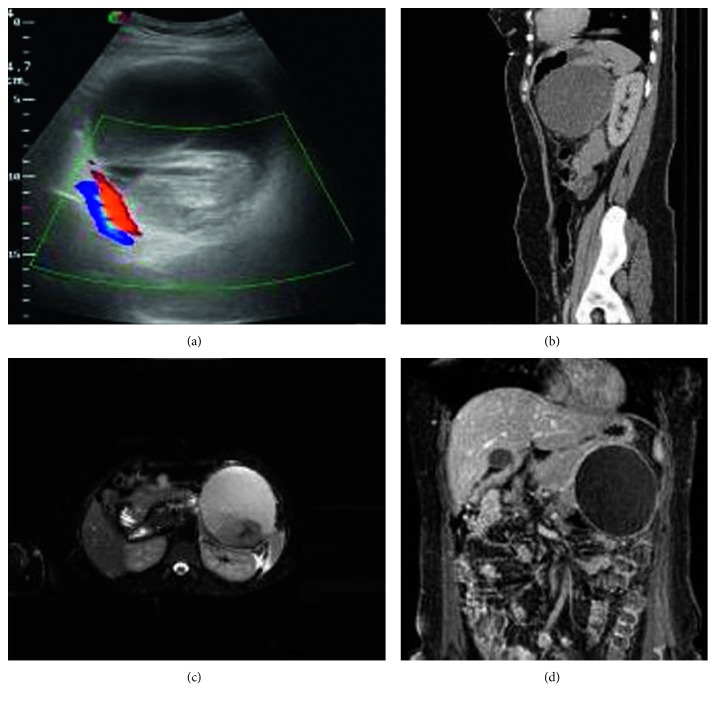
(a–d) Ultrasound imaging examination.

Laboratory results, including cancer biological markers, liver function and renal tests, and serum hormone profile (cortisol, aldosterone, calcium, urinary catecholamines, 5-HIAA, and metanephrine*s*), were within normal limits. During ultrasound examination of the abdomen, an anechoic lesion near the left kidney was revealed. The lesion contains echogenic mass and material consistent with a hemorrhagic cyst. Due to the location of the lesion, further imaging was carried out with CT and MRI. CT examination revealed a hypodense cystic lesion containing hyperdense material in close proximity with the left kidney and the tail of the pancreas, while T2-weighted sequences in MRI imaging with fat suppression showed high signal intensity within the mass. Areas of medium signal intensity with capsular enhancement of the lesion were also showed. Differential diagnosis was unclear, ranging between an adrenal lesion and a pancreatic lesion (Figure 1).

**Figure 2:**
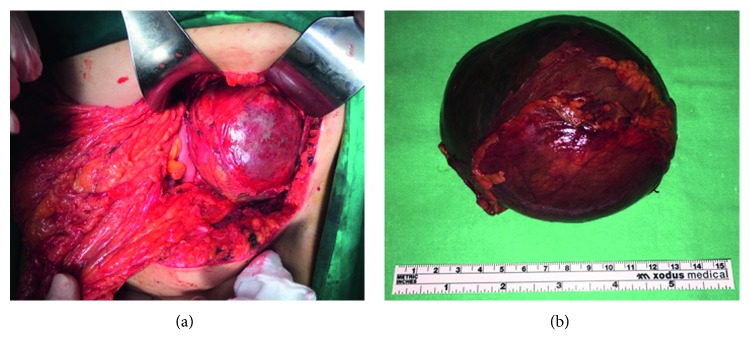
(a) Intraoperative findings. (b) Final specimen after surgical resection.

**Figure 3:**
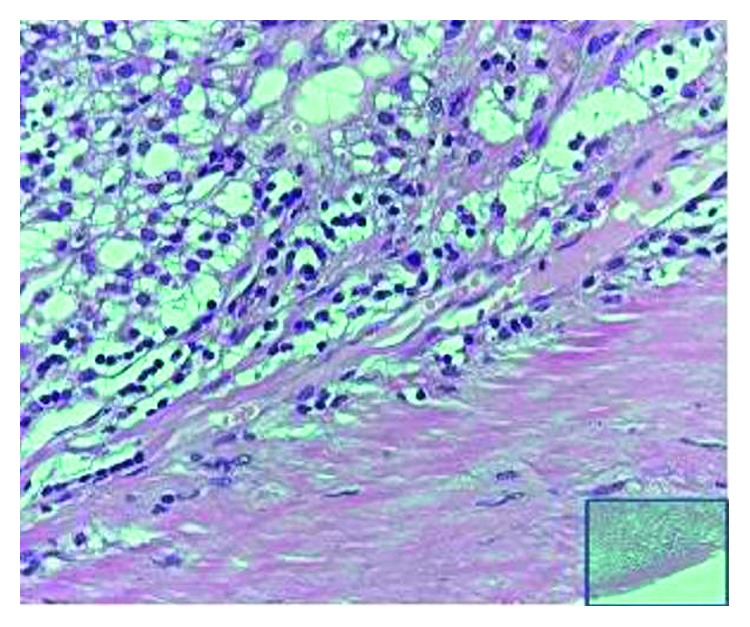
Histological examination (Hematoxilina eosina X60).

**Figure 4:**
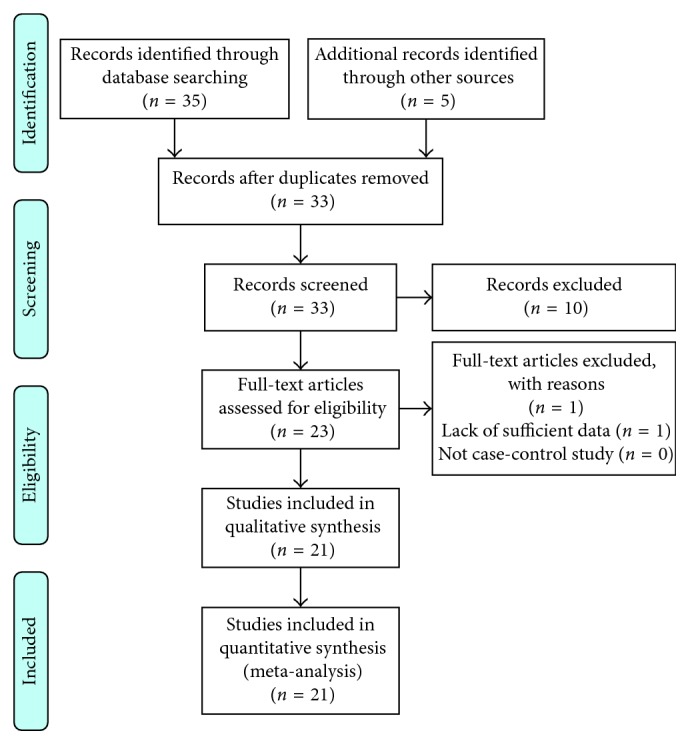
Prisma chart.

Surgical approach through left Kocher incision was decided, after thorough information and the patient's consent. A unilocular mass sized 11.7 × 9.3 × 6.6 cm with 1.0430 cm^3^ total volume was revealed in close relevance to the pancreas but with the origin from the left adrenal gland and was excised within healthy borders and sent for further pathologic examination (Figures 2(a) and 2(b)). The postoperative period was uneventful, and the patient was discharged on the sixth postoperative day. Pathological examination showed that the wall of the cyst is composed of the fibrous tissue without epithelial lining and massive hemorrhagic content. The normal adrenal tissue was found to be pericapsule. Histopathological findings confirmed a diagnosis of an adrenal pseudocyst (Figure 3). Three months later, in the first follow-up, the patient was found with good clinical condition, and laboratory tests and CT imaging were normal. In order to evaluate the treatment strategy of adrenal cysts in the last decade, bibliographic research through PubMed, Scopus, and Embase was performed. The search terms employed were “huge adrenal pseudocyst” and “adrenal pseudotumour.” From 33 initially published articles in the period 2006–2017, we spotted 21 fully documentated cases according to PRISMA Statement guidelines (Figure 4). 16 articles were in English, 2 articles were in Chinese, 2 were in Spanish, and 1 article was in German. Descriptive statistics were used appropriately. Means, medians, and standard deviations were used. Statistical analysis was performed with SPSS version 23 (SPSS Inc., Chicago, IL).

**Figure 5:**
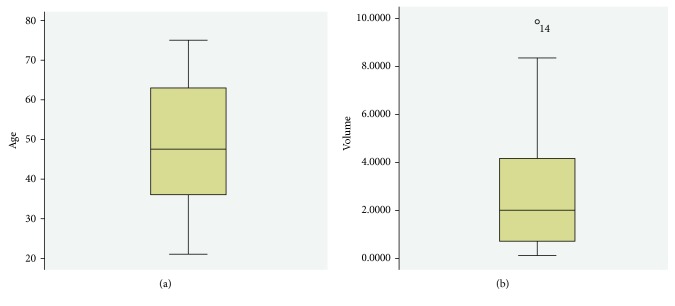
(a) Box plot diagram showing the median age of patients appearing with adrenal pseudocysts. (b) Box plot diagram of the mean volume of the mass in patients with adrenal pseudocysts.

Based on statistic analysis, it becomes clear that most adrenal pseudocysts are asymptomatic because of their small size and their location in the retroperitoneum; however, when they grow up significantly, they may cause compression of neighboring structures or/and increase intra-abdominal pressure [9]. The three most frequently reported clinical features are a dull pain in the anatomic area where the adrenal pseudocyst is located, gastrointestinal symptoms, and a palpable mass, while adrenalectomy, laparoscopic or open, is the preferable treatment method (Table 1). The ratio between men and women was 1  :  1.2, while the median age appears to be 47.5 (SD 16.699; *P*=0.200) (Figure 5(a)). The median volume of pseudocysts was estimated to be 2.8126 cm^3^ ranging from 0.1250 cm^3^ to 9.86 cm^3^ (SD 2.7555; *P*=0.200) (Figure 5(b)).

## 3. Discussion

### 3.1. History

Although pseudocysts represent the most frequently identified adrenal masses, endothelial cysts are found for up to 45% in autopsy series [1, 6, 8]. The autopsy series, probably, report a more accurate incidence of the different subtypes of adrenal cysts, since the surgical series take into account only patients who exhibited clinically significant symptoms, thus underestimating the true incidence of the various subtypes [1, 8]. Therefore, a realistic estimation of the subtypes of adrenal cysts is rather as follows: endothelial cysts 45%, pseudocysts 39%, epithelial cysts 9%, and parasitic (hydatid) cysts 7% [6].

Origins of adrenal pseudocysts still are a matter of debate. Some authors suggest that these lesions result from an intra-adrenal hemorrhage caused by trauma, a sepsis event, or any such systemic insult [10, 11]. It has also been speculated that the malformation of an artery or a vein which undergoes cystic dilatation or hemorrhage may result in the pathogenesis of a pseudocyst [10, 11]. Another theory suggests that such lesions are derived from true cysts that have lost their inner lining due to inflammation and/or bleeding within the cyst [10, 11]. Predisposing factors for adrenal hemorrhage, including trauma to the abdomen, infection, anoxia in infants, hemorrhagic diathesis, administration of antiplatelets or anticoagulants, embolism, and finally aneurysm, are reported in 32% of adrenal cysts [10–12]. There have also been reported at least 12 pregnant women with a diagnosis of adrenal pseudocysts, and a possible role of estrogens in their pathogenesis has been postulated [13]. However, there is not sufficient evidence to substantiate this theory, even if that fact would partly explain the predilection of adrenal cysts towards the female sex in general [13].

### 3.2. Clinical Features and Epidemiology

Adrenal pseudocysts may also be functional, causing syndromes like adrenal hypofunction (Cushing's syndrome) [14, 15]. Pseudocysts may also, occasionally, present with acute abdomen, hypovolemia, or a painful mass, when intracystic hemorrhage, rupture, or infection occurs [15]. Concerning the incident of spontaneous intracystic hemorrhage, this has been especially linked with pregnant women, since it has been proposed that high estrogen levels may cause the pseudocyst's rapid growth as well as its wall's relaxation [16]. In our case, pseudocyst was presented in a young age woman, with upper abdominal pain and a palpable mass without any other symptoms, and without history of pregnancy.

### 3.3. Differential Diagnosis

The most worrisome feature of adrenal cystic neoplasms, including the pseudocysts, is a reported incidence of malignancy estimated at 7% of the cases, especially if the pseudocyst's size exceeds 6 cm [6, 17]. An underlying carcinoma should be suspected and sought in patients with a high erythrocyte sedimentation rate, mixed echoes on ultrasonography, stippled calcification on plain films, and neovascularization on arteriography [18]. When trying to decipher the origin of a patient's symptoms, imaging techniques are utilized early rather than late in the diagnostic process. Concerning the cystic lesions of the adrenal glands, there may be some specific features that may be helpful in differentiating these from other space-occupying masses in US, CT, MRI, and nuclear medicine imaging.

US is usually the first imaging exam that may be employed for the evaluation of patients with a suspected adrenal mass, since it is a modality that has low cost and no exposure to radiation and allows for no confinement of claustrophobic or agitated patients. Nevertheless, the sensitivity of US for the detection of adrenal lesions is highly variable, with reported values from 66.7% to more than 90%, regardless of the lesion's size. Recently, contrast-enhanced US (CEUS), although not widely utilized, has also been added to the imaging armamentarium, as it has been reported to be able to detect or disprove the enhancement of the lesion's walls, thus accordingly pointing to a malignant or benign lesion [10].

CT characteristics of the adrenal pseudocyst include a well-determinated cyst that does not enhance with intravenous contrast, while the density of the cyst's content measures at water density or much higher, depending on the amount of the encased hemorrhagic debris [4]. Another useful differentiating CT feature is the thickness of the cyst's wall. The presence of calcifications also suggests a benign nature of the depicted cystic lesion [19].

MRI features of adrenal pseudocysts include a solid component with low intensity on both T1- and T2-weighted images and a second, smaller, fluid component. Complicated or hemorrhagic adrenal cysts may demonstrate variable signal intensity and may be difficult to differentiate from solid neoplasms. A limitation of MRI is, however, the poor sensitivity for detection of calcifications [4].

Nuclear medicine imaging, such as ^131^I-MIBG scintigraphy, may be used for the differentiation of an adrenal pseudocyst from a cystic pheochromocytoma, apart from the pertinent biochemical investigations, which should consist of a comprehensive preoperative hormonal and functional evaluation (serum vanyl-mandelic acid, adrenocorticotropic hormone, and cortisol levels) [20].

The differential diagnosis of an adrenal pseudocyst essentially includes all the upper abdominal space-occupying lesions, such as splenic, hepatic, and renal cysts, as well as mesenteric or retroperitoneal cysts, urachal cysts, and solid adrenal tumours [9, 12, 13, 16]. However, it must be noted that a preoperative diagnosis of a large adrenal pseudocyst can be very difficult indeed, due to the indistinct boundaries with surrounding organs as well as the presence of adhesions to neighboring organs [9], as in our case, where differential diagnosis of the adrenal or pancreatic origin of the mass could not be established through radiologic examination.

In excised specimens, the exact nature will be determined also with the aid of immunochemistry, as the adrenal pseudocyst's wall will contain adrenal cortical tissues, confirmed by A103 (Melan-A). Furthermore, a strong expression of factor VIII-related antigen, laminin, and CD34 will be apparent, in the absence of epithelial membrane antigen or keratin expression, suggesting additionally a vascular origin of these lesions [21, 22].

The treatment of choice for adrenal pseudocysts depends on several factors. According to the current treatment management, it is suggested that surgical excision should be done in all adrenal lesions > 5 cm, suspicious of malignancy, and functioning adrenal pseudocysts. However, it is recommended the excision of lesions even smaller, for example, 4 cm in size, if suspicious of malignancy. In lesions < 4 cm, a repeat CT scan 3 months later is usually advised, with a follow-up period at least for 18 months [23]. Laparoscopic excision permits small incisions, minimizes bowel manipulation, and decreases preoperative morbidity, leading to shorter hospital stay and faster recovery. Except for the general contraindications of laparoscopic procedures (unacceptable cardiopulmonary risk, uncorrected coagulopathy, abdominal sepsis, and intestinal obstruction), the absolute contraindication of laparoscopic adrenal surgery is the suspicion of a large adrenal cortical carcinoma, since open procedures allow better vascular control [13]. Other possible procedures for simple cysts, such as unroofing, open or laparoscopic, and percutaneous needle aspiration, were also referred [24].

## 4. Conclusion

Adrenal pseudocysts are rare lesions that may cause infrequent morbidity. Symptoms are usually atypical and depend on the pseudocyst size. Since the reported symptoms are vague due to the location of these lesions, a high suspicion index of the surgeon is required for the prompt diagnosis as well as suitable laboratory studies and imaging techniques. However, it has to be mentioned that the final diagnosis will be provided by pathology examination of the final specimen after the procedure. There are various ways that these cysts may be managed, which depend on the characteristics of the lesions, the surgeon's experience, and local resources, while the main treatment strategy remains the same in the last decade.

## Figures and Tables

**Table 1:** 

Author name	Year	Gender	Calcification	Age	Hemorrhage	Size	Volume	Procedure	Side
Paramythiotis	2017	Female	No	25	Yes	11.7 × 9.3 × 6.6	1.043	Pseudocyst removal	Left
Alvaro Martinez Manzano	2017	Female	Yes	73	Yes	5 × 5 cm	0.125	Open adrenalectomy	Right
Geleit	2016	Male	No	33	Yes	Large	1.848	Open adrenalectomy	Right
Pathaik	2015	Male	No	50	Yes	21 × 18 × 15	5.67	Open adrenalectomy	Right
Schrank	2014	Female	Yes	62	Yes	9.1 × 7.4 × 6.9	1.145	Laparoscopic adrenalectomy	Left
Soroush	2013	Female	No	32	Yes	15.1 × 11.5 × 17	2.95	Open adrenalectomy	Left
Angelico	2013	Female	No	30	Yes	10 × 7 × 10	0.7	Laparoscopic cyst removal	Left
Cantisani	2013	Male	No	75	Yes	19 × 14 × 12	3.192	Open adrenalectomy	Right
Passoni	2013	Male	Yes	69	Yes	12 × 9 × 7	0.756	Open adrenalectomy + splenectomy + pancreatic tail resection	Left
Mahmodlou	2011	Female	No	21	Yes	15 × 9.7 × 15	2.182	Open adrenalectomy	Right
Zanghi	2012	Female	No	39	Yes	8 × 8	0.448	Laparoscopic adrenalectomy	Left
Karim	2016	Male	No	70	Yes	22 × 20 × 19	8.36	Pseudocyst open removal	Right
Gupt	2011	Male	No	58	Yes	23 × 15 × 12	4.14	Open adrenalectomy	Left
Ujam	2011	Female	No	39	Yes	29 × 20 × 17	9.86	Laparoscopic adrenalectomy	Right
Marwah	2011	Male	No	60	Yes	6 × 6	0.252	Cyst removal	Left
Yusuharu	2011	Female	No	43	Yes	22 × 13 × 3	0.858	Cyst removal	Left
Momiyama	2011	Male	No	52	Yes	18 × 18	5.508	Open adrenalectomy	Right
Salemis	2011	Male	No	45	Yes	15.5 × 13.5	2.511	Laparoscopic adrenalectomy	Right
Wilkinson	2011	Female	No	64	Yes	12 × 6	0.504	Cyst unroofing	Left
Karaman	2011	Female	No	40	Yes	20 × 15	4.2	Open adrenalectomy	Left
Kim	2009	Female	No	38	Yes	9 × 9	0.729	Laparoscopic adrenalectomy	Left

## References

[1] Wedmid A., Palese M. (2010). Diagnosis and treatment of the adrenal cyst. *Current Urology Reports*.

[2] Bellantone R., Ferrante A., Raffaelli M., Boscherini M., Lombardi C. P., Crucitti F. (1998). Adrenal cystic lesions: report of 12 surgically treated cases and review of the literature. *Journal of Endocrinological Investigation*.

[3] Aloraifi F., O‘Brien G., Broe P. (2008). Giant adrenal pseudocyst treated laparoscopically: case report and review of the literature. *Open Surgery Journal*.

[4] Sivasankar A., Jeswanth S., Johnson M. A. (2006). Acute hemorrhage into adrenal pseudocyst presenting with shock: diagnostic dilemmas-report of three cases and review of literature. *TSW Urology*.

[5] Levison P. (1933). A case of bilateral adrenal cyst. *Endocrinology*.

[6] Foster D. G. (1966). Adrenal cysts. Review of literature and report of case. *Archives of Surgery*.

[7] Demir A., Tanidir Y., Kaya H., Turkeri L. N. (2006). A giant adrenal pseudocyst: case report and review of the literature. *International Urology and Nephrology*.

[8] Sebastiano C., Zhao X., Deng F.-M., Das K. (2013). Cystic lesions of the adrenal gland: our experience over the last 20 years. *Human Pathology*.

[9] Momiyama M., Matsuo K., Yoshida K. (2011). A giant adrenal pseudocyst presenting with right hypochondralgia and fever: a case report. *Journal of Medical Case Reports*.

[10] Cantisani V., Petramala L., Ricci P. (2013). A giant hemorragic adrenal pseudocyst: contrast-enhanced examination (CEUS) and computed tomography (CT) features. *European Review for Medical and Pharmacological Sciences*.

[11] Stimac G., Katusic J., Sucic M. (2008). A giant hemorrhagic adrenal pseudocyst. Case report. *Medical Principles and Practice*.

[12] Marwah S., Marwah N., Garg S., Mathur S. K. (2011). Adrenal pseudocyst mimicking cystic neoplasm of pancreatic tail. *Clinical Journal of Gastroenterology*.

[13] Angelico R., Ciangola C. I., Mascagni P., Manzia T. M., Colizza S. (2013). Laparoscopic adrenalectomy for haemorrhagic adrenal pseudocyst discovered during pregnancy: report of a case. *Surgical Laparoscopy, Endoscopy & Percutaneous Techniques*.

[14] Schrank Y., Madeira M. (2014). Massive hemorrhagic adrenal pseudocyst. *American Journal of Medicine*.

[15] Kim S. B., Joo H. S., Choi I. S., Song Y. J. (2009). Laparoscopic resection of an adrenal pseudocyst mimicking a retroperitoneal mucinous cystic neoplasm. *World Journal of Gastroenterology*.

[16] Papaziogas B., Katsikas B., Psaralexis K. (2006). Adrenal pseudocyst presenting as acute abdomen during pregnancy. *Acta Chirurgica Belgica*.

[17] Ujam B. A., Peters J. C., Tadrous J. P., Webster J. J., Steer K., Martinez-Isla A. (2011). Adrenal pseudocyst: diagnosis and laparoscopic management-a case report. *International Journal of Surgery Case Reports*.

[18] Mohan H., Aggarwal R., Tahlan A., Bawa A. S., Ahluwalia M. (2003). Giant adrenal pseudocyst mimicking a malignant lesion. *Canadian Journal of Surgery*.

[19] Johnson T. P., Horton M. K., Fishman K. E. (2009). Adrenal imaging with MDCT: nonneoplastic disease. *American Journal of Roentgenology*.

[20] Kyoda Y., Tanaka T., Maeda T., Masumori N., Tsukamoto T. (2013). Adrenal hemorrhagic pseudocyst as the differential diagnosis of pheochromocytoma–a review of the clinical features in cases with radiographically diagnosed pheochromocytoma. *Journal of Endocrinological Investigation*.

[21] Patnaik S., Htut A., Wang P., Eisenberg D., Miick R., Feyssa E. (2015). All those liver masses are not necessarily from the liver: a case of a giant adrenal pseudocyst mimicking a hepatic cyst. *American Journal of Case Reports*.

[22] Erem C., Celik F., Reis A., Hacıhasanoglu A., Gör A. (2005). Large adrenal pseudocyst presenting with epigastric distress and abdominal distention. *Medical Principles and Practice*.

[23] Passoni S., Regusci L., Peloni G., Brenna M., Fasolini F. (2013). A giant adrenal pseudocyst mimicking an adrenal cancer: case report and review of the literature. *Urologia Internationalis*.

[24] Salemis S. N., Nisotakis K. (2011). Giant adrenal pseudocyst: laparoscopic management. *ANZ Journal of Surgery*.

