# Corrigendum to “Surgical Management of a Giant Adrenal Pseudocyst: A Case Report and Review of the Literature in the Last Decade”

**DOI:** 10.1155/2018/6531610

**Published:** 2018-05-15

**Authors:** Daniel Paramythiotis, Petros Bangeas, Anestis Karakatsanis, Patroklos Goulas, Irini Nikolaou, Vasileios Rafailidis, Konstantinos Kouskouras, Vasileios Papadopoulos, Sofia Lypiridou, Georgia Karayannopoulou, Antonios Michalopoulos

**Affiliations:** ^1^1st Propedeutic Surgical Department, AHEPA University Hospital, Aristotle University of Thessaloniki, Thessaloniki, Greece; ^2^Department of Radiology, AHEPA University Hospital of Thessaloniki, Thessaloniki, Greece; ^3^Department of Pathology, AHEPA University Hospital of Thessaloniki, Thessaloniki, Greece

In the article titled “Surgical Management of a Giant Adrenal Pseudocyst: A Case Report and Review of the Literature in the Last Decade” [1], there was an error in the legend of Figure 1, which should be corrected as follows:

## Figures and Tables

**Figure 1:**
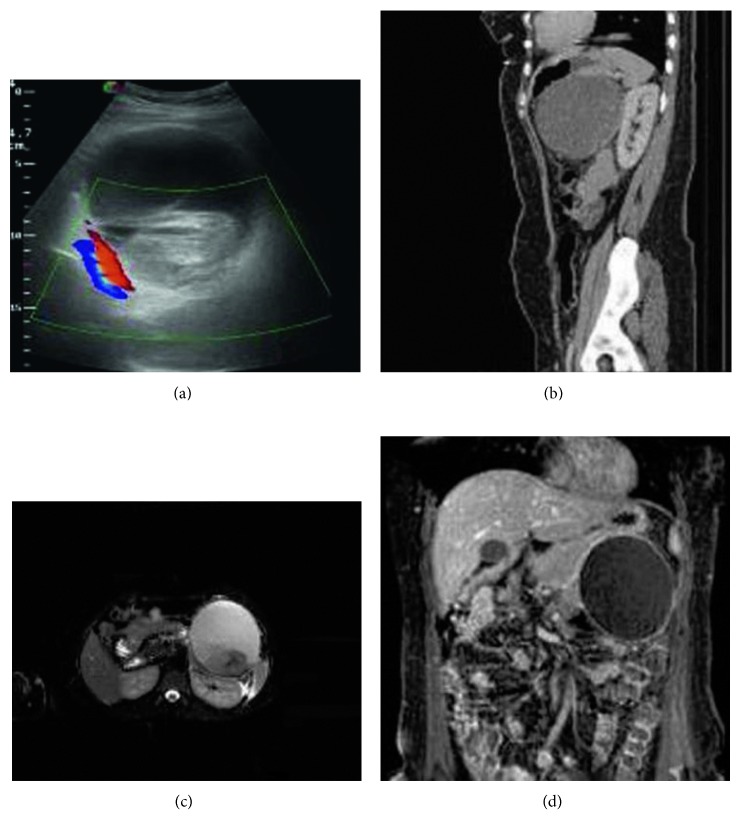
(a) Colour Doppler ultrasound image showing a cystic lesion containing echogenic debris with no vascularity. (b) Sagittal contrast-enhanced CT image showing the cystic lesion located within the left kidney and the tail of the pancreas. (c) Axial T2-weighted MR image with fat suppression confirming the cystic nature of the lesion. Note: it is made of dependent low-signal intensity debris, in keeping with hemorrhagic content. (d) Coronal contrast-enhanced T1-weighted MR image showing only mild capsular enhancement but no enhancement within the cyst.
